# Application of Bioelectrochemical Systems and Anaerobic Additives in Wastewater Treatment: A Conceptual Review

**DOI:** 10.3390/ijms24054753

**Published:** 2023-03-01

**Authors:** Nhlanganiso Ivan Madondo, Sudesh Rathilal, Babatunde Femi Bakare, Emmanuel Kweinor Tetteh

**Affiliations:** 1 Green Engineering Research Group, Department of Chemical Engineering, Faculty of Engineering and The Built Environment, Durban University of Technology, Steve Biko Campus, S4 Level 1, Durban 4000, South Africa; 2 Environmental Pollution and Remediation Research Group, Department of Chemical Engineering, Faculty of Engineering, Mangosuthu University of Technology, Durban 4026, South Africa

**Keywords:** anaerobic digestion, bioelectrochemical systems, anaerobic additives, sewage sludge, wastewater treatment

## Abstract

The interspecies electron transfer (IET) between microbes and archaea is the key to how the anaerobic digestion process performs. However, renewable energy technology that utilizes the application of a bioelectrochemical system together with anaerobic additives such as magnetite-nanoparticles can promote both direct interspecies electron transfer (DIET) as well as indirect interspecies electron transfer (IIET). This has several advantages, including higher removal of toxic pollutants present in municipal wastewater, higher biomass to renewable energy conversion, and greater electrochemical efficiencies. This review explores the synergistic influence of bioelectrochemical systems and anaerobic additives on the anaerobic digestion of complex substrates such as sewage sludge. The review discussions present the mechanisms and limitations of the conventional anaerobic digestion process. In addition, the applicability of additives in syntrophic, metabolic, catalytic, enzymatic, and cation exchange activities of the anaerobic digestion process are highlighted. The synergistic effect of bio-additives and operational factors of the bioelectrochemical system is explored. It is elucidated that a bioelectrochemical system coupled with nanomaterial additives can increase biogas–methane potential compared to anaerobic digestion. Therefore, the prospects of a bioelectrochemical system for wastewater require research attention.

## 1. Introduction

The over-dependence on energy that relies on the usage of fossil fuels, the production of greenhouse gases negatively affecting the environment, and the escalation in fossil fuel prices have pushed investigators nowadays to search for other techniques to produce sustainable energy. Vast growth in population and urbanization has also resulted in the generation of enormous quantities of waste, resulting from inadequate treatment and management [1,2]. The search for renewable energy is even now a requirement to accommodate the ever-rising demand for energy and the decrease in reliance on fossil fuels [3]. All at once, there is a quest for renewable energy techniques that are environmentally friendly and cost-effective, for instance, hydro, wind, solar, and bioenergy techniques [4,5].

Anaerobic digestion is a promising renewable energy process (bioenergy) since it generates biogas, which consists chiefly of methane that can be used for the generation of electricity [6,7]. There is a quest for improving the efficiency of the conventional anaerobic process, primarily because the process has high instability, low activity of microbes, low contaminant removal rates, and low methane generation [8,9]. Recently, a method that utilizes the combination of an anaerobic digestion process with an electromagnetic field has been found to improve microbial activity as well as reduce toxic pollutants existing in wastewater [10,11]. The most promising electromagnetic field technique is the bioelectrochemical system, where electrochemically-active microorganisms, also called exoelectrogens, improve the redox mechanisms at the surface of the electrode. A bioelectrochemical system has earned more interest in anaerobic digestion since the utilization of these two techniques promotes the degradation of biochemical matter in wastewater [12]. Although promising, the bioelectrochemical system is still very new to fully understand its role in anaerobic digestion [13]. The impact of electromagnetic fields, such as bioelectrochemical systems, on electrode kinetics, is regarded as the most debatable at present [14]. On the other hand, the process control indicators affecting the bioelectrochemical system, such as temperature, electrochemical efficiencies, and external resistance have to be properly controlled, correctly designed, and well-studied to optimize the efficiency of the system, enhance the stability of the system, and thus avoid inhibition.

**Figure 1:**
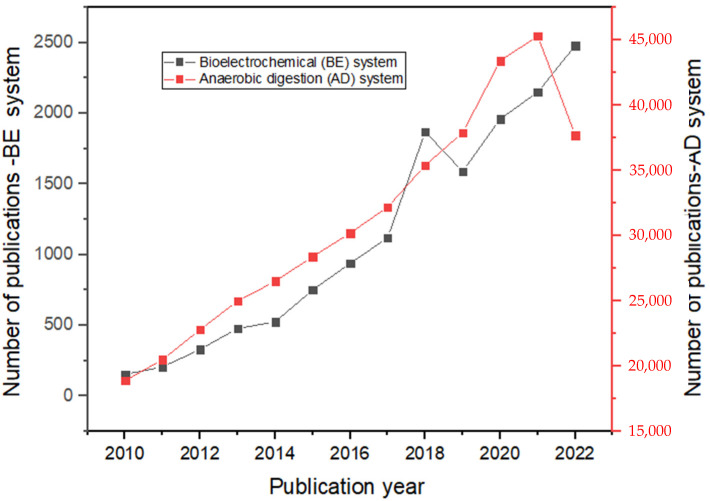
Google scholar publication trend of bioelectrochemical and anaerobic digestion systems from 2010–2022.

**Figure 2:**
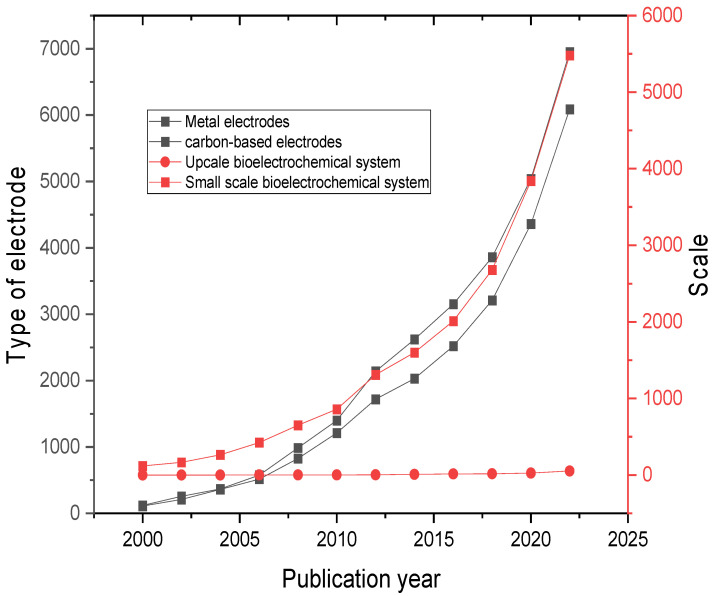
Google scholar publication trend of type of electrode and scale from 2000–2022.

The supplementation of anaerobic additives (such as magnetite-nanoparticles) in a bioelectrochemical system is an attractive new concept that may denote a realistic approach to trigger a bioelectrochemical system created for the anaerobic treatment of wastewater and enhance the efficiency of exoelectrogenic substrate oxidation systems. There have been very few studies on the synergistic use of magnetite-nanoparticles and bioelectrochemical systems in wastewater treatment. Nonetheless, the few studies that have reported on the combination of these systems have shown major improvement over the traditional anaerobic digestion process [11,15,16,17]. Cruz Viggi et al. [18] sprinkled magnetite-nanoparticles in an electrochemically-active biofilm with the support of a magnetic field and found that the electron transfer of microorganisms away from the electrode was highly improved when the magnetite-nanoparticles were positioned only at the electrode/biofilm interface. Despite such promising findings, the effect of bioelectrochemical processes and anaerobic additives on anaerobic digestion has been inadequately investigated (Figure 1); more studies are needed to fully understand the redox reactions that take place at the surface of the electrode. More studies are focusing on carbon-based electrodes as opposed to metal-based electrodes (Figure 2). Moreover, more studies have focused on small-scale bioelectrochemical systems as opposed to large-scale/upscale bioelectrochemical systems.

As some of the limitations of the traditional anaerobic digestion processes have been discussed, this review concentrates on the option for the biochemical system and anaerobic additives to maximize the treatment of wastewater for biogas production. The paper first gives a brief background of the traditional anaerobic process, leading to the limitations of this process. Then, anaerobic additives are proposed as a way of improving the performance of the traditional method. Thereafter, the use of a bioelectrochemical system is explored as an alternative option for improving the efficiency of traditional anaerobic digestion. Lastly, the synergistic use of both anaerobic additives and a bioelectrochemical system is discussed and recommended as a promising concept for the treatment of wastewater for biogas production.

## 2. Anaerobic Digestion

The method that is perhaps the most efficient and most promising in the treatment of some biochemical wastes, as well as wastewater and complex solid wastes, is anaerobic digestion, mainly because of the number of advantages it has compared to the other methods [19]. Advantages of the anaerobic digestion process include reduction of greenhouse gas emissions, conversion of carbon dioxide energy to methane, accommodation of high loading rates, and removal of pathogens [20,21,22]. Due to these benefits, the anaerobic digestion process is, thus, worth further investigation.

Anaerobic digestion involves the degradation of a biodegradable substance, such as sewage sludge by anaerobic microorganisms in an oxygen-free system [23]. The microorganisms feed on the organic matter, thereby converting it into biogas that is predominated by an energy-containing substance, methane, as well as carbon dioxide [24,25,26,27,28,29]. In an oxygen-free environment, anaerobic digestion can convert almost 90% of the energy kept in the biological matter into methane. The 10% of the biological matter that remains in wastewater is firstly dewatered and then disposed of. The anaerobic process is an effective method for several wastewater products, for instance, agricultural, industrial, and municipal wastewater.

### 2.1. Biochemical Mechanisms Found in Anaerobic Digestion

**Figure 3:**
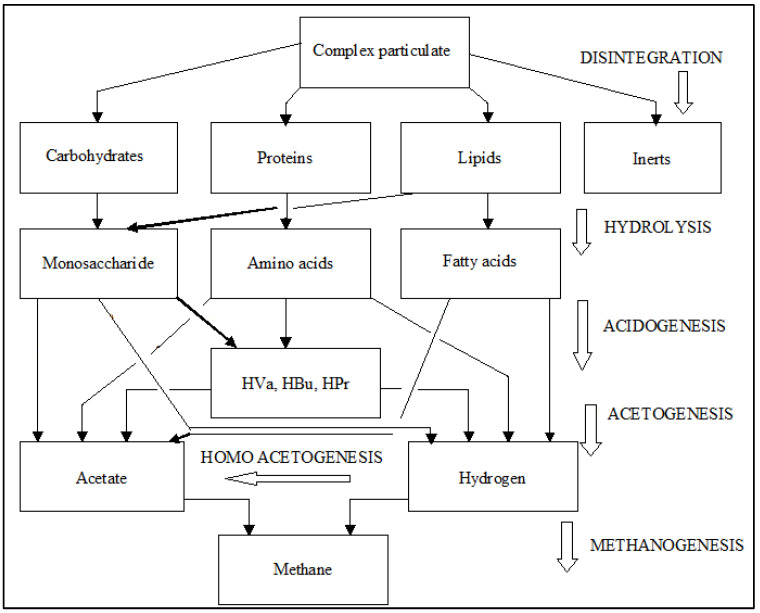
The anaerobic microbiological process diagram [31].

The overall digestion process involves both microbiological and physiochemical mechanisms, where the product of one stage is used as a feedstock for the subsequent stage. In detail, the process of anaerobic treatment usually involves the hydrolysis stage, acidogenesis stage, acetogenesis stage, and methanogenesis stage as illustrated in Figure 3 [30]. The hydrolysis stage is the only stage that includes physiochemical mechanisms (i.e., that involves the use of extracellular enzymes), and the other stages involve microbiological mechanisms (i.e., it involves the use of intracellular enzymes).

Wastewater such as sewage sludge normally entails complex constituents, namely carbohydrates, proteins, and lipids [32]. These biochemical constituents are not easily biodegraded by anaerobic microbes which means that they have to be broken down into smaller size molecules that are enough to be taken by microbes. This is accomplished in the hydrolysis stage, where lipids, proteins, and carbohydrates are firstly immersed into the liquid and are biodegraded by extracellular microorganisms, namely lipases, proteases, and cellulases into long-chain fatty acids, amino acids, and saccharides, respectively.

The hydrolysis stage is normally the rate-limiting stage for complex constituents, as this stage determines the total quantity of matter available for the subsequent stage and, hence, affects the movement rate of the entire anaerobic digestion process [33,34]. Generally, pre-treatment techniques are used by most researchers to enhance the hydrolysis stage, therefore, decreasing the hydraulic time of the digestion process as well as improving biogas production. These pre-treatment techniques can either be thermal-related or chemical-related. The latter includes the supplementation of lime, ammonia, or acid, whereas the former involves the use of steam or hot water [35,36,37].

The acidogenesis stage is the first stage of fermentation and continues the degradation of products from the hydrolysis stage by acidogenesis microorganisms (acidogenic), thereby, generating many organic substrates together with ammonia, hydrogen, and carbon dioxide. This stage is energetic and normally the fastest stage in the whole anaerobic process.

The acetogenesis stage is very important for the successful production of methanogenesis products. This stage involves the biodegradation of volatile fatty acids (VFAs) by acetogenesis microorganisms, known as acetogens, into methane, acetate, carbon dioxide, hydrogen, and water. Almost all reactions taking place in the acetogenesis stage have a positive change in Gibbs’s energy. This means that the overall mechanism involved in this stage is an endothermic one. Endothermic systems in anaerobic digestion are only accomplished in syntrophic conditions, a process that takes place between acetogens and hydrogenotrophic methanogens [38].

The last stage in anaerobic digestion is the methanogenesis stage, where intermediate products that were obtained in the process are transformed by methanogens into biogas [39]. This stage is achieved by methane-forming microorganisms. Any of the three bacterial pathways can be used to produce methane in the methanogenesis stage, namely methylotrophic, acetoclastic cleavage of acetic acid, and hydrogenotrophic methanogens. All of these pathways occur simultaneously in the methanogenesis stage. In a proper working digester, approximately 72% of the produced methane takes the acetoclastic cleavage of the acetic acid pathway. The remaining 28% takes the hydrogenotrophic pathway, whereas a small amount is produced via the methylotrophic pathway.

### 2.2. Limitations of the Traditional Anaerobic Digestion Process

Biochemical activities, for instance, syntrophy, metabolism, catalysis, and enzyme activities control the effectiveness of the digestion process for enhanced methane production. Perhaps, the most significant factor that may hinder biochemical activities and, hence, methane production is inhibition. The nature and type of biochemical activities and mechanisms affect the stability of the anaerobic process. Anaerobic digestion has certain inherent problems depending on the type of feed and anaerobic factors, for example, mass transfer that is slow especially when the solid content is high, VFA build-up, imbalanced carbon-to-nitrogen ratio, the obstinacy of lignocellulosic deposits, inhibition due to ammonia, sulphur, and insufficient micronutrients (see Table 1) [40,41]. Such anaerobic problems may at times result in slow methanogenic activity along with low methane yield as well as hindering the bacterial community of the anaerobic digestion system.

## 3. Additives Used in Anaerobic Digestion

Various methods are implemented in anaerobic digestion to reduce the effect of inhibition and enhance stability to enhance the production of methane. This includes optimizing the concentration of solids to improve mass transfer, maintaining pH in the digester by adding a buffer, co-digesting substrates to balance the carbon-to-nitrogen ratio, and pre-treating to break the lignin structure to enhance the methanogenic stage.

Alternatively, the addition of several materials as support substrates to the digestion process offers an efficiently feasible answer for the above-mentioned problems of anaerobic digestion for improved anaerobic stability [51]. This includes (1) high electrical conductivity material to enhance syntrophic action, (2) small amounts of metals for metabolism enhancement, (3) nanoparticles to enhance metabolism and catalytic action, (4) microorganisms to enhance enzymatic activity, and (5) surface-active material to enhance cation exchange action [40,52].

### 3.1. Syntrophic Activity

Even though the rate-limiting stage differs and depends on the kind of feed, the syntrophy activity is usually regarded as a key parameter that greatly governs the entire rate of anaerobic digestion [19]. In a digester, a well-adjusted syntrophy involving the acidogenic stage and methanogenic stage presents a thermodynamically stable digestion system that is for the conversion of VFAs. The process of converting VFA to intermediate products such as acetate, butyrate, lactate, and ethanol as a result of syntrophy microorganisms stimulates methanogenesis microorganisms to digest the intermediate products as substances for CH_4_ production accompanied by reacting H_2_/CO_2_.

On the other hand, carbonaceous additives with high electrical conductivity promote the syntrophy action involving acid production by acidogenesis microorganisms and CH_4_-producing microorganisms by interspecies electron transfer (IET), a concept that involves direct interspecies electron transfer (DIET) and indirect interspecies electron transfer (IIET) [53,54,55,56]. This process is indicated in Table 2. In digesters, hydrogen and formic acid can simply be digested by methanogenesis microorganisms to generate CH_4_ using IIET on the condition that partial pressure is favorable to IIET. The phenomenon of syntrophy can become hindered if the hydrogen partial pressure is big, which might interrupt the IIET process, resulting in the volatile fatty acid build-up and the anaerobic digestion system becoming unstable. The DIET process, in contrast, once it occurs in the digestion process, can substitute hydrogen as the sole path of IET in the digester and assists in maintaining syntrophy action linking acetogenesis and methanogenesis microorganisms [57].

The DIET process, employing conductive substrates, is by far the most effective method for the generation of methane when compared to IET using electron carriers for instance, hydrogen, a key path of methane generation in traditional anaerobic digestion [64]. In the DIET process, the electron produced by the microorganisms is immediately taken by electron-receiving microbes as a result of networks formed by electrical conductive nanowires and cytochrome produced by microorganisms. Moreover, the process can assist in providing an ideally useful path because it has a higher negative Gibbs value (∆G_0_), and metabolites do not need to generate and diffuse [19].

**Figure 4:**
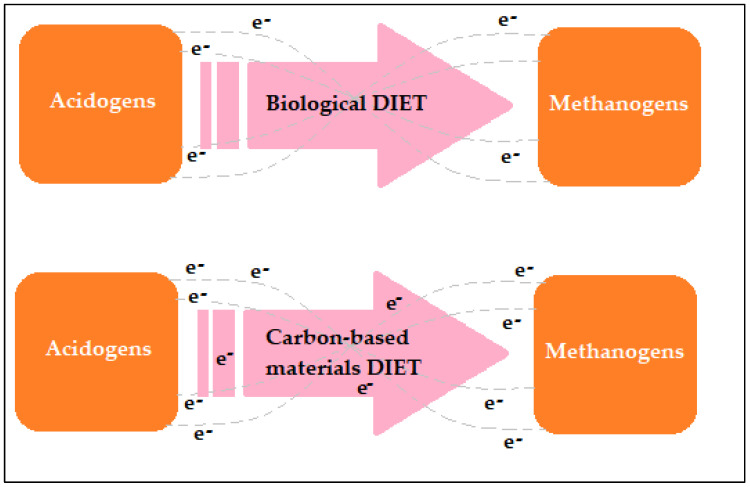
Biological and carbon-based direct interspecies electron transfer (DIET) methods. Adapted from [40].

Furthermore, the supplementation of conductive substrates to the digestion process can assist in promoting the DIET method for microorganisms that are unable to produce nanowires for IET (see Figure 4). The rate of movement of the IET in the DIET system is about a million times higher than that in the IIET process. Therefore, the inability in the digestion system to be stable as a result of volatile fatty acids build-up at a higher loading rate can be enhanced by the DIET process. Moreover, carbonaceous conductive substrates, for instance, bio-char, carbon cloth, activated carbon, granular activated carbon, and magnetite, may operate to improve the DIET process in the digestion process as they assist in the acceleration of the volatile fatty acids build-up and present substances to methanogenesis microorganisms. Many investigators found a helpful effect on the methanogenic stage as well as stability when adding such additives for the anaerobic digestion of biochemical products and wastes [40].

The utilization of biochar made by the thermal degradation of food waste as an additive to anaerobic digestion was extensively investigated by Shin et al. [65]. Adding 1% of food-waste biochar to the digester enhanced biogas generation by almost 10% and the content of methane by approximately 4%. Furthermore, the researchers discovered that biochar behaved like a medium with trace elements that encouraged the proliferation of microorganisms and enhanced the performance of anaerobic digestion. Feng et al. [66] investigated the influence of adding carbon cloth on the anaerobic digestion of biochemical wastewater at different mixing rates. The study revealed that the generation of methane can be enhanced by 10.1 to 23.0% and the efficiency of chemical oxygen demand (COD) removal was increased by 14.6% upon the addition of carbon cloth in the digester with no mixing at organic loading rates in the range of 2.1–4.2 g of COD per L-day. On the other hand, the improvement influence was only seen when the organic loading rate was very high (i.e., 4.2 g of COD per L-day) in a perfectly mixed reactor. Mostafa et al. [67] studied the effect of adding magnetite-nanoparticles and carbon nanotubes on anaerobic digestion. Both additions increased methane generation, and its favourable effect improved when the concentration of oleic acid was increased. The ultimate enhancements of 114% and 165% in comparison with the control were obtained by magnetite-nanoparticles and carbon nanotubes, respectively, at oleic acid of 4 g of COD per L. The excretion of electron shuttles, such as substances that are like protein and humic, were discovered to be enhanced by adding magnetite nanoparticles and carbon nanotubes. 

### 3.2. Metabolic Activity

**Figure 5:**
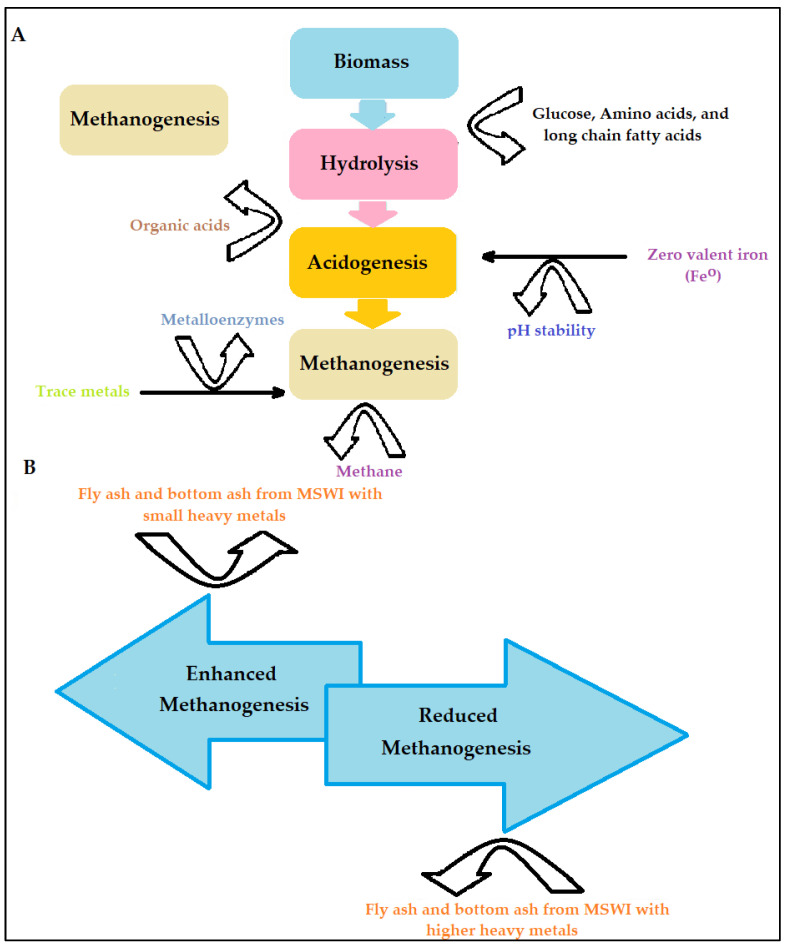
(**A**) Mechanisms of metal-based additives in anaerobic digestion. (**B**) Municipal solid waste incineration ash as additive in anaerobic digestion. Adapted from [40].

It is worth noting that, despite the potential toxic effect of metals, nearly all of them are required in anaerobic digestion for structural purposes, optimum growth, and optimal performance [68,69]. Small amounts of metals, an additive to anaerobic digestion, may be useful for the metabolism enhancement of bacterial cells (Figure 5). The use of substances, for instance, nickel, cobalt, iron, molybdenum, and other trace metals improve the metabolic activity of methanogenesis microorganisms, resulting in high methane production.

Most enzymes need metals as co-factors for their roles in anaerobic digestion. Perhaps the most plentiful metal present in cells is iron [68]. Because nearly all metalloenzymes found in the route of the generation of biogas have several clusters of Fe_4_S_4_, Fe_3_S_4_, or Fe_2_S_2_, iron is vital for cytochromes and the generation of methane [68,69]. Moreover, iron is an economical trace metal for enhancing methane production and the stability of the process. The process of adding iron in anaerobic digestion regularly promotes methane generation by extending the peak of the gas generation and increasing the activities of cellulase [69]. For instance, Bakari et al. [70] studied the influence of iron (F^0^), both steel wool and scrap iron, on the anaerobic digestion of sewage sludge. The outcome of this study revealed that: (a) steel wool performed better than scrap iron on the removals of chemical oxygen demand (COD) and phosphates (b) the optimum dosage for the removals of nutrients and the biochemical matter was 10 g/L scrap iron (c) the least removed contaminant was nitrogen (d) highest removals of COD and phosphates were 88.0%, and 98.0%, respectively. Therefore, iron-supported anaerobic digestion substantially removed the nutrients and biochemical matter from domestic sewage.

### 3.3. Catalytic Activity

Elements of nanoscale dimensions are known as nanoparticles and range from around 1 to 100 nm [71]. The antibacterial properties, enormous energy-storing capacity, and the capability to present higher surface area (surface/volume ratio) have raised researchers’ attention to these particles in bioenergy usages. As a result of the ability of these substrates to improve catalytic capability within a big surface area for mechanisms, the nano-additives have furthermore acquired significant attention in biochemical energy.

**Figure 6:**
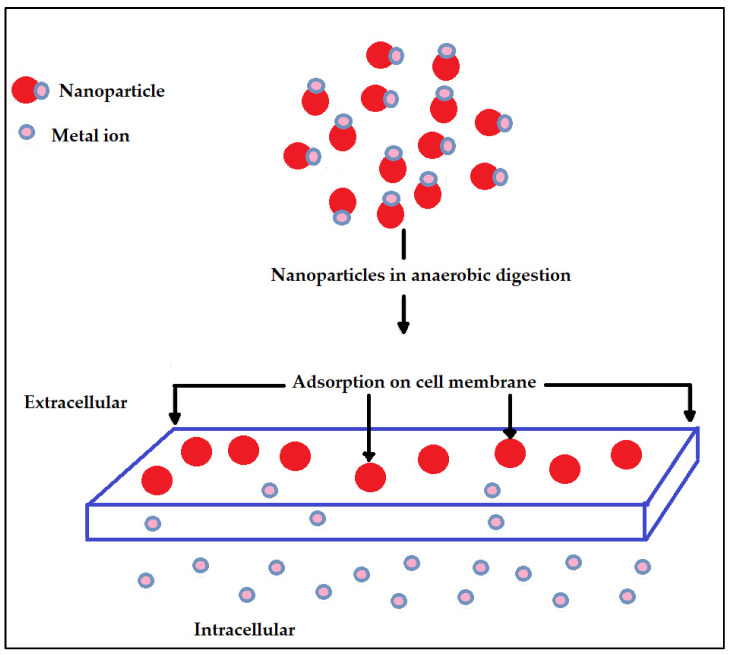
Mechanism of nanoparticles addition in anaerobic digestion [40].

Adding nanoparticles in anaerobic digestion (Figure 6) and also the effect of these nanoparticles on methane generation has been investigated quite often by many researchers over the past few years. Nanoparticles are presently used for detecting and removing biochemical materials including metals, algae (for example, toxins of cyanobacteria), nutrients (for example, nitrate, ammonia, and phosphate), carbon-based substances, virus, cyanide, microbes, antibiotics, and parasites. Essentially, four types of nanomaterials are currently used as useful materials for wastewater treatment purposes, namely metal oxide nanoparticles, carbonaceous nanoparticles, zero-valent nanoparticles, and dendrimers [40,71]. Córdova-Lizama et al. [72] studied the influence of cobalt and iron zero valent nanoparticles on the anaerobic digestion of waste-activated sludge. The outcome revealed that zero-valent iron nanoparticles and cobalt nanoparticles enhanced the early stages of the anaerobic digestion of waste-activated sludge. The highest hydrogen productions were found to be 5.40 and 5.74 mLH_2_/g volatile solids added (VS_added_) for cobalt nanoparticles and zero-valent iron nanoparticles, respectively. 

García et al. [73] investigated the influence of titanium dioxide, cerium dioxide, gold, and silver nanoparticles on the activity of bacterial communities proposed for the treatment of wastewater. The outcome of the study showed that cerium dioxide nanoparticles resulted in the highest inhibition in the generation of biogas (almost 100%) and a great inhibitory action of other biochemical matters; silver nanoparticles resulted in an intermediate inhibition in generation of biogas (in the range 33–50%) and a small inhibition in the action of other biochemical matters, and titanium dioxide and gold nanoparticles resulted in only small or no inhibition for all investigated biochemical matters.

Chhetri et al. [74] developed a novel nanotechnology-based method (flocculation-based magnetic nanoparticles) to enhance the quality of groundwater. The outcome of the study demonstrated the prospective of magnetic nanosponges to enhance the groundwater quality and support the development of an economical greatest management technique (biochemical methane potential) that also uses customary coagulants at concentrated animal feeding operation and other wastewater treatment plants. The study showed that the coagulation process was improved by the usage of magnetic nanosponges, which can enhance the formation of flocs rapidly and efficiently with the ability to totally gravitate in the sedimentation tanks. The utilization of magnetic nanoparticles together with coagulants led to a higher reduction in the turbidity and total organic carbon of dairy farm wastewater in comparison with swine lagoon water, which indicated that magnetic nanosponges are more effective in enhancing the quality of water for highly contaminated lagoons.

Award et al. [75] explored the use of ultrafiltration membranes for wastewater treatment. The study also investigated the effect of the composition and type of the hydrophilic additives of nanoparticles (for example, titanium dioxide, zinc oxide, graphene oxide, etc.). It was suggested that amongst all kinds of membrane methods, ultrafiltration is regarded as a useful separation system and purification system. It was suggested that it is usually used to treat oily wastewater with <20 μm oil droplet size and <400 ppm oil content. Furthermore, another method has been tried to enhance the performance of polymeric membranes with a valuable influence by using additives, for instance, hydrophilic polymers, inorganic nanoparticles, and grafted and amphiphilic copolymers. Common inorganic particles that have been widely utilized to fabricate membranes are silicon dioxide, titanium dioxide, aluminum dioxide, magnesium oxide, graphene oxide, etc. Their use in membranes has considerably increased their antifouling properties with regards to oil products.

Kaegi et al. [76] studied the behavior of metallic silver nanoparticles in a pilot wastewater treatment system supplied with municipal wastewater. Silver nanoparticles were spiked into the non-aerated tank. Silver concentrations verified by inductively coupled plasma–mass spectrometry agreed well with estimates based on mass balance considerations. Analyses of a transmission electron microscopy (TEM) confirmed that nanoscale silver particles were absorbed to wastewater biosolids, both in the effluent and in the sludge. During the initial pulse spike, freely dispersed nanoscale silver particles were only seen in the effluent. X-ray absorption spectroscopy analyses showed that most silver in the effluent and in the sludge existed as silver sulphide. The outcome from the experimental works revealed that silver nanoparticles conversion to silver sulphide took place in the non-aerated tank within 2 h. Chemical and physical transformations of silver nanoparticles in wastewater treatment plants control the fate, the transport and also the bioavailability and the toxicity of silver nanoparticles and, thus, have to be taken into consideration in future risk assessments.

### 3.4. Enzymatic Activity

Preparing enzymes or adding microbes as a means of substituting the physiochemical pretreatments performed before anaerobic digestion has been studied in-depth recently. However, adding enzymes or microbes instantaneously in anaerobic digestion has not gained much attention. When anaerobic digestion is enhanced as a result of microorganisms, the process is called bioaugmentation [77]. The composition of the microbial population has an absolute influence on how the anaerobic digestion process behaves as well as its products since the performance of all biochemical mechanisms of anaerobic digestion rely on the movement of the microorganisms secreting essential enzymes, as well as the cellulosome multi-enzyme complexes, that are attached to the substance cell surface, therefore resulting in cellulosic hydrolysis.

Thus, how the anaerobic digestion system performs can be controlled by the manipulation or enrichment of the population of microbes existing in the reactors. Once the anaerobic system fails to succeed, mainly because of the bacterial population shifts, bioaugmentation with fresh microorganisms that have been added may be performed to restore the satisfactory performance of the anaerobic system. Such microbial shifts can take place when the system is stressed as well as in transitional phases, for instance, temperature variations and a decrease in pH. In such instances, the greater performance of anaerobic digestion is achievable by improving the population of microbes or the addition of microorganisms with new abilities.

However, the vital step for attaining a large quantity of designated bacterial community is selecting an inoculum as a source of microbes and the amount of inoculum to be used. Investigators usually give emphasis to the establishment of a methanogenesis population in the early phase of the anaerobic digestion system to accomplish a stable system. The large quantity of archaea responsible for the methanogenic stage is regarded as a crucial aspect in improving methane generation. Thus, most investigations focus on the methanogenesis population to stabilize the anaerobic digestion system in its early phases.

**Figure 7:**
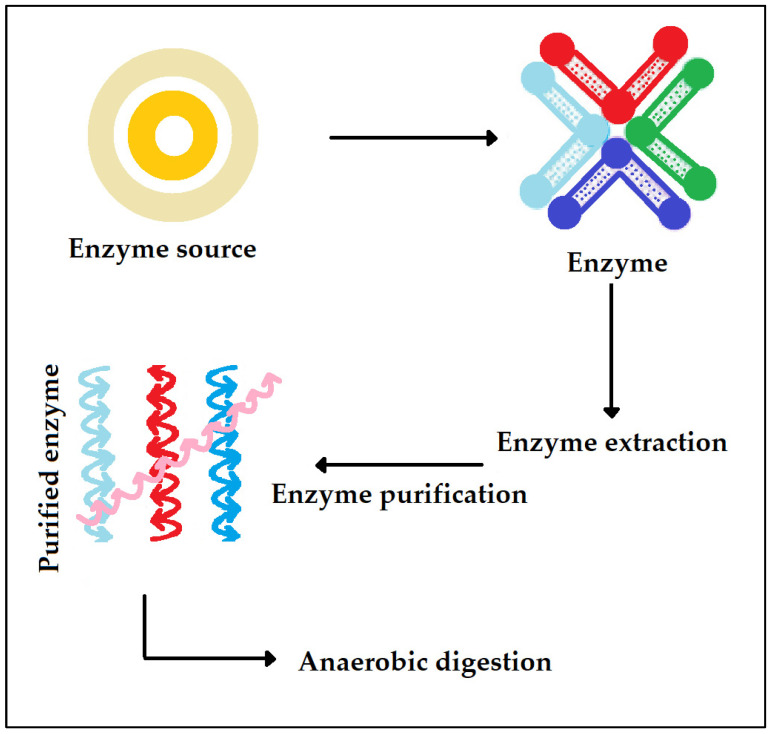
Extraction of enzyme for application in anaerobic digestion.

Another technique that may be used for augmenting the anaerobic digestion system is by adding enzyme preparations in digesters instead of microbes (Figure 7). Earlier studies showed enhanced methane generation from the pretreatment of lignocellulosic biomass including commercial and crude enzymes. However, enzymes can also be utilized for biomass treatment instantaneously in the anaerobic digestion process [78,79]. Enzyme addition in the anaerobic digestion process can be of great benefit because it is capable of withstanding a large range of salinity, pH, and temperature and is capable of avoiding unfavorable situations that cause inhibition in microbial activity. Enzyme addition rather than ordinary pretreatment techniques will, moreover, help to prevent the undesirable impact of produced inhibitory products on the activities of enzymes. Phenols as well as furfural produced in the pretreatment stage of the anaerobic digestion process have been shown to result in inhibition in the cellulosome activity. Furthermore, the better freedom of movement and the small size allow the enzymes to obtain greater access to the feed in comparison to microbes.

Aside from the addition of enzymes, biochemical substances can, moreover, be introduced in the anaerobic process to enhance the activity of enzymes. With regards to this, the process of adding carbon-based acids with negatively charged ions such as lactate, formate, and acetate have been found to function like promoters to improve the cellulosome activity for the duration of anaerobic digestion within 50, 100 and 200 mM concentrations, respectively. Above these values, the activity of cellulosomal enzymes is inhibited [80].

### 3.5. Cation Exchange Activity

**Figure 8:**
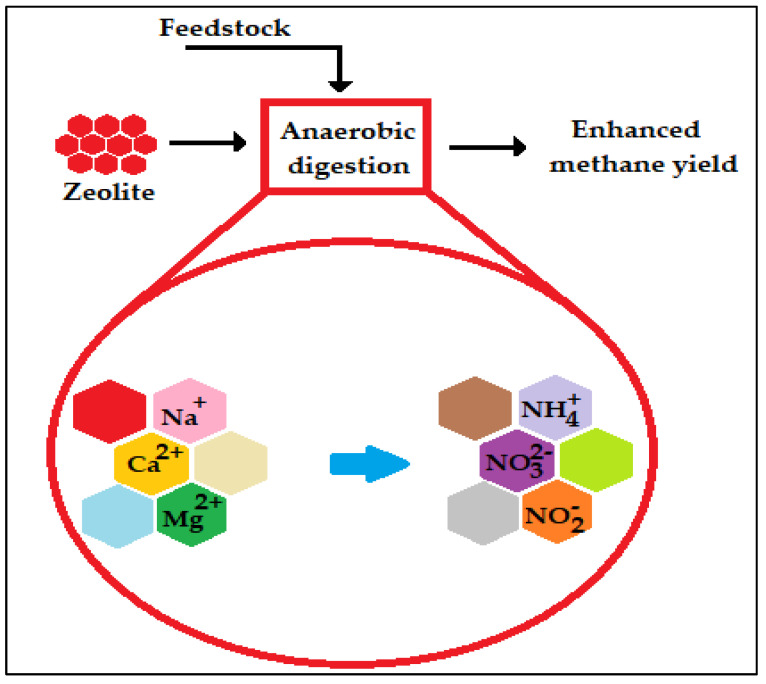
Cation exchange activity of zeolites [40].

Surface-active substances, for instance, zeolites, may affect the bacterial community and their conversion in the digestion process. In addition to this, owing to the porous structure of zeolite might assist in anaerobic digestion if immobilization of organic matter is needed [81,82]. Zeolite, with its favorable features for adhering microorganisms, has also been usually used in anaerobic digestion as an ion exchanger for removing ammonium because of the existence of Mg^2+^, Ca^2+^, and Na^+^ positive ions in its crystal structure (Figure 8). Zeolites improve the ammonia/ammonium equilibrium in anaerobic digestion and the probability of decreasing ammonia and ammonium ions in water/wastewater [83]. This characteristic feature is also very significant for improving the performance of the anaerobic systems when treating wastewater with very high contents of nitrogen compounds, for example, poultry waste, because it avoids ammonia inhibition.

A study was conducted by Wang et al. [84] on the impacts of zeolites on the anaerobic digestion process of ammonium-rich swine wastes. The use of zeolites increased the composition of methane by 19.7% and the overall methane yield by 120.9 CH_4_/kg VS compared to the absence of zeolites.

Essentially, zeolite is used as a surface where microorganisms can grow, as a result decreasing the lag phase and at the same time improving production in a specified period [83]. Hansson [85] investigated the effect of the zeolite-clinoptilolite in a continuous anaerobic digester. The results indicated a substantially smaller build-up of VFAs in comparison with a control digester at an organic loading rate of 4.8 kg VS/(m^3^·day) and a hydraulic retention time (HRT) of 30 days. The same observation was found upon the addition of zeolites in a batch reactor, which also revealed a declined lag phase.

However, the cation exchange characteristics of zeolite, makes this substrate not desirable for the solid-state anaerobic process. Stress can be made utilizing zeolite in dry anaerobic digestion of slaughterhouses, sludge, and food waste since these substrates contain high content of nitrogen.

Table 3 shows a summary of various additives used in anaerobic digestion.

## 4. Bioelectrochemical System

To effectively direct anaerobic digestion mechanisms to completion and to prevent high volatile acid build-up, for example, propionic acid and butyric acid, the physiobiological interaction between various microbes as well as methanogens should be increased [86]. This interaction (i.e., the concept of DIET) can be improved by combining methods, for instance, supplying a conductive or additive material, applying an external voltage and the anaerobic digestion process [19,41]. The application of an external voltage is a new concept in anaerobic digestion. This process is otherwise known as the bioelectrochemical process combined with the digestion process and was originally used for the formation of hydrogen. In this process, the generated hydrogen is oxidized to methane on the cathode, whereas the rate of the total methane generated is enhanced [19].

### 4.1. Utilization of Bioelectrochemical System on Anaerobic Digestion

**Figure 9:**
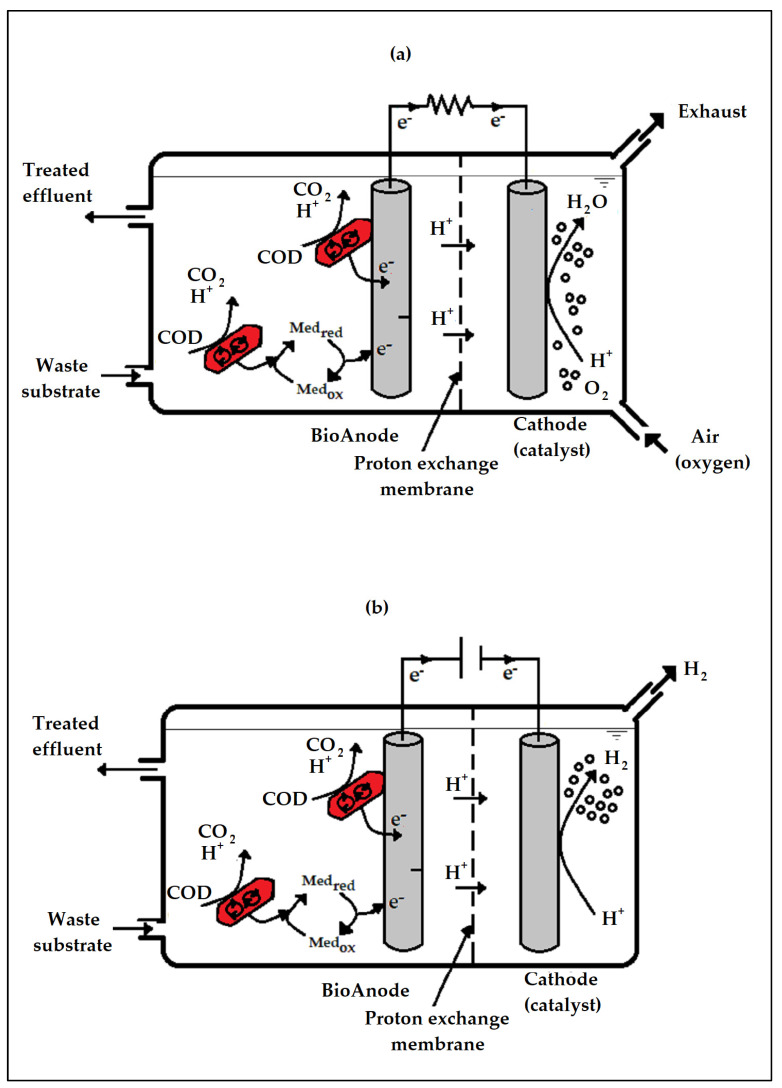
Schematic diagram of: (**a**) a MFC; and (**b**) a MEC. Adapted from [94].

The bioelectrochemical system utilizes microbes attached to the electrodes to catalyze the reduction and/or oxidation mechanisms (Figure 9). A bioelectrochemical system is known as a microbial fuel cell (MFC) when electricity from the degradation of organic substances is generated, and a microbial electrolysis cell (MEC) when electrical energy is introduced via the external circuit to encourage non-thermodynamically favorable mechanisms [87,88]. Both the MFC and MEC have gained interest as very useful technologies for the production of energy and chemical transporters [89].

In the anodic section of an MFC, the biochemical substrate undergoes oxidation as a result of bacterial communities, together with exoelectrogenic communities, otherwise known as electro-active microorganisms [90]. The electrons produced (e^−^) in this reaction are not transported to a soluble terminal receiver of electrons (CO_2_, Fe^3+^, ${\mathrm{SO}}_{4}^{2-}$ and O_2_), however, they are transferred to the anodic compartment (insoluble electron receiver outside of microbial cells). The transfer can take place via constituents linked with the membrane and via nanowires of bacterial origin or soluble electron mediators [91,92]. From the anodic compartment, electrons (e^−^) are directed through a circuit located externally, joined to an external resistance, to the cathodic compartment, where the electrons are reduced during the conversion of O_2_ to produce H_2_O. The charge balance between the anode and cathode electrodes is retained since there is an instantaneous flow of ions (cations, for instance, K) across the ion exchange membrane. The change in voltage across the anodic and cathodic electrodes is called an electromotive force (EMF) and is the main reason for the electrons to flow [93]. The system is not fully effective, because, in the bacterial population, the concepts of fermentation and respiration of microbes are competing for the e^−^ of the biochemical matter. The effectiveness of a MFC is highly governed by the losses of energy of the mechanisms represented as overpotentials, and to which extent e^−^ generated are converted to the required product, represented as coulombic efficiency [87]. However, in addition to the generation of energy, another advantage of the MFCs and MECs can as well be the recovery or treatment of toxic pollutants, namely sulphates, sulphides, and nitrates.

**Figure 10:**
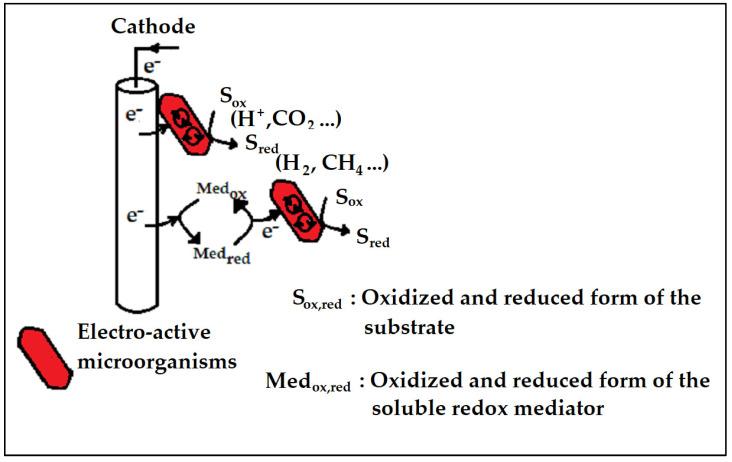
Schematic diagram of the mechanism generated in a bio-cathode. Adapted from [94].

In a MEC, as is the case with a MFC, electrochemically active microbes (exoelectrogens) utilize the anode as a terminal electron receiver for the biochemical matter degradation up to CO_2_ whilst at the same time discharging protons to the wastewater [95]. The electron (e^−^) moves from the anodic compartment to the cathodic compartment through an exterior circuit whereas the protons migrate to the cathodic section across a membrane that separates the electrodes [96,97]. At the cathodic compartment, in the existence of an appropriate (bio)-catalyst, a soluble electron receiver combines with electrons (Figure 10), producing a desired product. The difference with a MFC is that a MEC needs to be powered with external voltage to make certain that the reaction at the cathodic compartment is thermodynamically favourable. With regards to the MEC, the main aim is to produce H_2_ or CH_4_ which, in this specific instance, are generated in the cathode [98].

To date, several substrates have been investigated as possible sources of energy for the generation of electricity in the MFC, such as amino acids, carbohydrates (for example, cellulose, starch, sucrose, and glucose), proteins, alcohols (for example, ethanol, and methanol), and volatile fatty acids—VFAs (for example, butyrate, acetate, and formate). The usage of biochemical wastewater is a fascinating alternative to using pure compounds, allowing residual flow treatment whilst producing renewable energy. Pant et al. [89] showed the ability to use an extensive range of complex substances to support the generation of electrical power in a MFC, for instance, swine wastewater, domestic wastewater, oxalate, wastewater from the generation of beer, or recycling of paper, or the anaerobic digestate. The agreement between the effluent of the traditional anaerobic digestion process and the influent of bioelectrochemical systems makes the MFC appropriate as an enhancing treatment process after the anaerobic digestion, or CH_4_ generating MEC to be a promising technique to obtain extra energy (which in this case is CH_4_) of the remaining biochemical substrate present in the digestate of the anaerobic digestion process.

Unlike the treatment of manure in anaerobic digestion, MFC results in the generation of electricity or power directly, and biogas, which comprises chiefly of CO_2_ and CH_4_. The MFC has potential benefits of operation and performance compared to other methods presently employed for the generation of electrical energy from a biochemical substrate. Firstly, the direct conversion of electricity present in the biochemical substrate theoretically results in great conversion efficiencies. Next, the MFC operates efficiently at atmospheric temperature as well as lower temperatures and is capable of treating low biochemical substance fluctuations. Finally, the MFC does not need subsequent biogas oxidation since it is chiefly made up of CO_2_ and not in CH_4_ or H_2_, and it typically has no valuable content of energy [99].

Even though originally the main focus of a bioelectrochemical system was its ability to generate an electrical current through the use of the MFC, the focus of the researchers is now shifting to other, more beneficial options. Recently, bioelectrochemical system techniques have gained much attention in anaerobic digestion, because the combination of the two processes can allow the recovery and/or production of valuable products such as hydrogen, industrial chemicals, bioelectricity, metals, and nutrients [100].

Hassanein et al. [101] investigated the influence of power generation and the treatment of waste when utilizing dairy manure in a combined MEC system and anaerobic digestion over the traditional anaerobic digestion with no external influence (i.e., control). Cumulative hydrogen and methane generation in the digester with anaerobic digestion and MEC (2430 mL hydrogen and 23,600 mL methane) was greater than the anaerobic control (0.00 mL hydrogen and 10,900 mL methane). An et al. [102] examined the effect of using a carbon-modified copper foam electrode on the bioelectrochemical methane generation from food waste. The highest methane generation in the bioelectrochemical system was 338.1 mL methane/L, which was substantially greater than the 181.0 mL methane/L of the control digester.

### 4.2. Factors Influencing the Efficiency of the Bioelectrochemical System

Several process control indicators affect the performance of a bioelectrochemical system. Parameters that influence the bioelectrochemical system include temperature, electrochemical efficiencies, and external resistance. Proper control and design of these parameters have to be achieved to optimize the system efficiency, improve the stability of the system and, therefore, avoid inhibition of the system.

#### 4.2.1. Temperature

Just like most anaerobic digesters, both MECs and MFCs are highly temperature-dependent systems. The generation of power in the MFC and the generation of hydrogen in the MEC, together with the removal of COD in both kinds of digesters, change significantly with a change in working temperature. Generally, temperature values that are either extremely high (for example, greater than 45 °C) or very low (for example, below 15 °C) drastically obstruct the generation of power in a MFC and hydrogen generation in the MEC, whereas temperature ranges from 30 to 40 °C appear to improve the way these digesters perform [103]. The greatest removal of COD in both MEC and MFC systems has been suggested to be between 25 and 35 °C. With regards to the principle of economy, Tang et al. [104] recommended the optimum temperature value of the MFC as 30 °C, although a MFC performs better at 35 °C. By the fitted results, the optimum temperature of the MEC was extremely nearly the same as that of the MFC, which indicates the possibility of combining these two kinds of systems under similar conditions.

The effect of temperature on MFC and MEC is a result of the diverse resulting activities of microbes. Other researchers have found the domination of electrogenic microbes [105], and improved activity and growth of electro-active biofilms [106] at the optimum values around 30 °C. The total activity of microbes declines when the temperature of the system becomes too high, which consecutively hinders the microbial formation of protons and electrons. Li et al. [107] studied the effect of temperature in the range of 10 to 55 °C on the generation of power of the MFC digester. The results suggested that an increase in temperature from 10 to 33 °C resulted in an improvement in current density and then it began to fall. On the other hand, an increase in temperature from 43 to 55 °C indicated no constant power.

One positive fact is that the variations of hydrogen production in a MEC and power generation in a MFC are typically below 10% when the temperature is in the range of 20 and 35 °C [108,109,110], which indicates that the two systems are capable of tolerating temperature variations across a broad span of temperature values in realistic situations for the treatment of wastewater. Heidrich et al. [109] found that the biofilms attached to the anodic and cathodic sections had self-heating influences that made them acclimatize to substandard temperature values. It must be noted that the utmost favorable temperature values for hydrogen production and power generation are different from the optimum temperature values for the removal of COD, probably for the reason that the microorganisms largely effective in degrading the biochemical substrates are frequently different from exoelectrogens, i.e., electrochemically active microorganisms.

#### 4.2.2. Electrochemical Efficiencies

The electrochemical efficiencies or properties, namely current density, power density, electric current, magnetic field, heterotrophic methane yield, electrical conductivity, electrochemical methane yield, and coulombic efficiency, are the key parameters in electrochemical systems because they provide an in-depth understanding of how ions behave in the anaerobic digestion process. The current density is probably the most fundamental parameter in electromagnetic field design such as bioelectrochemical systems. It may be defined as the overall electrical current that flows across a unit cross-sectional area; Equation (1) may be used to obtain the current density (j) of a bioelectrochemical system. Current density affects the electrochemical oxidation of a bioelectrochemical system as it regulates the ability for active radical generation on the electrode surface [111]. Although the performance of a bioelectrochemical system is highly favored at higher current densities, the value should not be too high. At very high current densities, concentration losses arise primarily as a result of an inadequate mass transfer by diffusing chemical substances to the electrode surface.

(1) $$j=\frac{I}{A}$$

where I denotes current in Amps (A), and A represents the anode cross-sectional area in m^2^.

Although CH_4_ is the gas of interest in anaerobic digestion, it is essential to know if the CH_4_ produced to take the autotrophic pathway or heterotrophic pathway. The autotrophic pathway, otherwise known as the hydrogenotrophic pathway, entails the generation of complex substances from constituents that are not complex (Equation (2)). On the other hand, heterotrophic or acetoclastic pathway entails the degradation of complex substances to constituents that are not complex (Equation (3)).

(2) $$4H_{2}+{\mathrm{CO}}_{2}\to {\mathrm{CH}}_{4}+2H_{2}O\;\Delta G=-135\;\mathrm{kJ}/\mathrm{mol}$$

(3) $${\mathrm{CH}}_{3}\mathrm{COOH}\to {\mathrm{CH}}_{4}+{\mathrm{CO}}_{2}\;\Delta G=-33\;\mathrm{kJ}/\mathrm{mol}$$

The electrochemical methane yield (EMY) and heterotrophic methane yield (HMY) are important electrochemical indicators since they assist in distinguishing the pathway that the produced CH_4_ take, in other words, the autotrophic pathway or the heterotrophic pathway. A process wherein both heterotrophic methanogenesis and electrochemical methanogenesis take place provides an increase to greater COD to CH_4_ conversion, and this takes place at EMY greater than 100% [112]. Equations (4) and (5) may be used to calculate the EMY and HMY, respectively [112].

(4) $$\mathrm{EMY}=\frac{\left\lfloor \frac{V_{M}}{22.450\;}\right\rfloor }{\left[\frac{n_{E}}{8\times F}\right]}\times 100\%$$

(5) $$\mathrm{HMY}=\frac{\left\lfloor \frac{V_{M}}{22.450\;}\right\rfloor }{\left[\frac{\left({\mathrm{COD}}_{F}-{\mathrm{COD}}_{D}\right)\times {\;V}_{F}}{64}\right]}\times 100\%$$

where $V_{M}$ represents CH_4_ accumulated in mL. The value $n_{E}$ denotes the total number of electrons (e^−^), which is calculated as the section under the current versus time graph, in other words, $n_{E}=\int I.\mathrm{dt}$. The term F represents Faraday’s constant and is 96.485 C/mol e^−^. The terms ${\mathrm{COD}}_{F}$ and ${\mathrm{COD}}_{D}$ represent the COD of the feed and COD of the digestate, respectively. The value $V_{F}$ represents the wastewater volume of the feed in mL.

The coulombic efficiency (CE) is another significant electrochemical parameter that shows a relationship between EMY and HMY. The CE is used to determine the overall electron recovery. More specifically, the parameter calculates the extent to which the e^−^ produced are converted to the desired product. The greater the CE value, the more effective the bioelectrochemical system.

Equation (6) can be used to determine the coulombic efficiency [112]:

(6) $$\mathrm{CE}=\frac{\mathrm{HMY}}{\mathrm{EMY}}\times 100\%=\frac{8\times n_{E}}{F\times V_{F}\times \left({\mathrm{COD}}_{F}-{\mathrm{COD}}_{D}\right)}\times 100\%$$

Another significant parameter is electrical conductivity which may be used in determining the flow of current or ions inside the bioelectrochemical system and not outside the bioelectrochemical system as is the case with current density.

Several researchers have studied the effect of electrochemical efficiencies on bioelectrochemical systems (Table 4).

#### 4.2.3. Influences of External Resistance on How a Bioelectrochemical System Performs

External resistance (R_external_) influences the way both MFC and MEC perform, as it limits the movement of electrons (e^−^) from the anodic compartment to the cathodic section [117]. In accordance with Ohm’s Law (V = R_external_ I), the current (I) and potential (V) outputs will be influenced. Consequently, according to the power output (W) equation, by W = R_external_ I^2^, the power output is likewise influenced. In bioelectrochemical systems, higher external resistance results in a decrease in both power density and treatment efficiency [103]. In general, the potential of the anode, which directly affects the availability of the anode as an electron receiver, is controlled by the external resistance. Consequently, the growing competition between the populations of microbes that are electrogenic and those that are non-electrogenic is affected when dissimilar external resistances are employed to the systems. Similarly, the growing competition amongst various electrochemically-active microorganisms is likewise affected, indirectly by the variation of the micro-environmental situations or directly by the utilization of an anode [117]. Ultimately, the bacterial population structures created under different external resistances would not be the same, therefore influencing the utilization of biochemical matter and the related formation of protons in the anodic section [118]. The build-up of protons in the anodic section would decrease the pH of the solution, which will consequently affect the environment of the biofilm. The population of microbes in the anodic biofilm is highly vulnerable to the variation of external resistance when the voltage of the anode is very small. For values below −1.5 V, the effects on many detected Geobacter species were found to be noticeable [118].

Many researchers have studied the effect of external resistance on bioelectrochemical systems. For instance, Mersinkova et al. [119] studied the effect of external resistance on the metabolic behaviour of biofilm of the anode electrode in MFC. The outcome of the study revealed that both too high and too low external resistance deteriorated the bio-electrochemistry of respiration of the anode electrode by moving the bacterial metabolism to usual substrate fermentation. The greatest conditions for respiration and efficient substrate mineralization of almost 70% were obtained in the microbial fuel cell digester with an external resistance of 100 Ω. Kamau et al. [120] investigated the influence of external resistance on electrochemical efficiencies in a MFC using cow dung. The outcome of the study showed that the greatest voltage of 0.153 V was found on day 7 at an external resistance of 33 kΩ. Moreover, power, current density, and power density ranges were 0.001–10 mW, 0.1–23.29 mA/m^2^, and 7.5 × 10^−7^–3.1036 mW/m^2^, respectively.

## 5. Synergistic Influence of the Bioelectrochemical System and Anaerobic Additives on Anaerobic Digestion

The addition of anaerobic additives in a bioelectrochemical system is a promising method that may represent a feasible approach to stimulate a bioelectrochemical system intended for the anaerobic digestion of wastewater and enhance the efficiency of exoelectrogenic substrate oxidation processes. There have been very few investigations on the supplementation of anaerobic additives in a bioelectrochemical system in wastewater treatment. Even so, the very few investigations that have been reported on the synergism of these techniques have shown major improvement over the conventional anaerobic digestion system. Most studies have recommended magnetite-nanoparticles as a promising anaerobic additive in the bioelectrochemical system since it assists the interspecies transfer between Archaea and microbes, with enhanced microbial diversity in the reactor [121,122].

**Figure 11:**
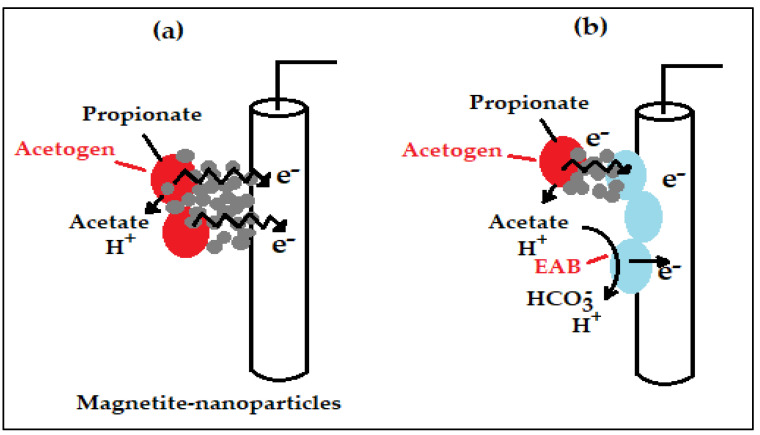
Proposed reactions supporting the influence of magnetite-nanoparticles on the degradation of propionate: (**a**) electron transfer that comes from oxidation of propionate to the electrode compartment via magnetite-nanoparticles, and (**b**) electron transfer that comes from the degradation of propionate to electrochemically-active bacteria through magnetite-nanoparticles [18].

Cruz Viggi et al. [18] examined the effect of magnetite-nanoparticles on the bioelectrochemical treatment of sewage sludge. It was found that, the existence of magnetite-nanoparticles had only small influences on acetate concentration profiles, it was probable that nanoparticles directly or indirectly influenced the electrons scavenging (and/or of molecular hydrogen) deriving from oxidation of propionate, by the reactions represented in Figure 11. In the first proposed reaction (Figure 11a), the electrons obtained from the oxidation of propionate are conveyed from the acetogenic bacteria directly to the electrode and, thus, avoiding the formation of intermediate molecular hydrogen, with the magnetite-nanoparticles acting as electron channels. In the second proposed reaction (Figure 11b), the electrons discharged from the oxidation of propionate are conveyed to electrochemically-active bacteria using an interspecies electron transfer reaction that is driven by magnetite. Eventually, electrochemically-active bacteria transfer the electrons that were obtained to the electrode compartment by utilizing it as the electrode respiratory electron receiver [123]. All in all, in both situations, magnetite-nanoparticles accelerate the bacterial population by utilizing the anode as a different basin for electrons obtained from the oxidation of propionate, thus, enhancing the degradation of substrate and generation of electrical current.

The use of an anaerobic electrochemical process with power supply is capable of transferring electrons outside the cells via exoelectrogens; this combination creates close interaction of several microbes for enhanced methane production via the hydrogenotrophic reactions, namely DIET (Equation (7)) and IIET (Equations (8) and (9)).

DIET pathway:

(7) $${\mathrm{CO}}_{2}+8H^{+}+8e^{-}\to {\mathrm{CH}}_{4}+2H_{2}O$$

IIET pathway:

(8) $$2H^{+}+2e^{-}\to H_{2}$$

(9) $${\mathrm{CO}}_{2}+4H_{2}\to {\mathrm{CH}}_{4}+2H_{2}O$$

A major discovery of the investigation by Cruz Viggi et al. [18] was the positive influence of magnetite-nanoparticles on the bioelectrochemical treatment of sewage. Comparable in certain respects to what was discovered when VFAs were used as the source of energy and carbon, the oxidation of propionate in synthetic sewage sludge was observed to be enhanced, which confirms the positive influence of magnetite-nanoparticles in accelerating the substrates and metabolites oxidations whose deterioration needs a syntrophy collaboration amongst bacteria. In this manner, probably, the same effect happened also for other, as yet not identified, synthetic sewage constituents, for example, proteins and lipids.

Nevertheless, it is worth noting that magnetite-nanoparticles facilitate the electron transfer to the electrode compartment by improving the conductivity of the bacterial biofilm that grows on the exterior of the electrode, and consecutively decreasing the resistance that ions and electrons encounter as they flow inside a bioelectrochemical system [124]. Outcomes of very recent findings by Madondo et al. [11] come in favor of this latter discovery: when the synergistic influence of the use of a bioelectrochemical system and magnetite-nanoparticles was investigated, the use of magnetite-nanoparticles improved the electrical conductivity of the bioelectrochemical system. In essence, electrical conductivity denotes the ability to transmit or conduct electrical flow in the solution, and the process must have thermodynamically satisfactory mechanisms. Equation (10) may be used to determine electrical conductivity (s):

(10) $$s=\frac{j}{E}=\frac{1}{r}$$

In this case, the term j denotes current density, the term E represents electric field intensity, and r represents resistivity. In a bioelectrochemical system, the term r (resistivity) represents resistance that ions/electrons encounter as they move within a bioelectrochemical system; in other words, this term represents the ohmic losses. Thus, from Equation (7), the electrical conductivity of a solution is inversely proportional to ohmic losses and directly proportional to the flow of current, which is represented by current density. Therefore, the high electrical conductivity of the magnetite-nanoparticles is likely to enhance the way the digester performs as it reduces the ohmic losses; the great concentration of ions and electrons in the bioelectrochemical system accelerates the flow of electrons in the exterior electrical circuit (current density) [125], which increases coulombic efficiency, improves biogas generation, and enhances the treatment of wastewater [11,15,16,17].

Madondo et al. [15] studied the synergistic influence of magnetite-nanoparticles and bioelectrochemical systems on the anaerobic digestion process, where four digesters were compared, specifically a MEC, MFC, MEC containing magnetite-nanoparticles, and a control digester. All digesters were fed with sewage sludge (0.5 L) and inoculum (0.3 mL). The digester containing magnetite was fed with 1 g of magnetite nanoparticles. The focus of the study was on the composition of methane and biogas production, electrochemical efficiencies, pH, and removal of pollutants. The MEC containing magnetite-nanoparticles revealed higher bacterial activity, enhanced methane composition (by 43% in comparison with 41% of the control), and decreased pollutants (COD, color, phosphates, turbidity, total organic carbon, and total suspended solids) by over 81.9%. In terms of electrochemical efficiencies, the same digester had the highest electrical conductivity of 275 µS/cm and a current density of 25.0 mA/m^2^.

Obviously from these findings, the synergistic application of magnetite-nanoparticles and the bioelectrochemical system seems very promising for wastewater treatment since it enhances the DIET and IIET [126]. However, the studies done so far have only focused on methane and biogas production, electrochemical efficiencies, stability indicators (i.e., pH), and toxic contaminant removals. The concept has recently been discovered, and as a result, there are so many gaps that need to be explored. The chemistry behind the synergistic application of magnetite-nanoparticles and the bioelectrochemical system is yet to be studied. Thus, more studies are required to fully understand the redox reactions that take place at the surface of the electrode to improve the efficiency of the anaerobic digestion process.

## 6. Conclusions

This review addresses limitations regarding the use of the traditional anaerobic digestion process, by proposing the use of anaerobic additives (such as syntrophic, metabolic, catalytic, enzymatic, and cation exchange activities) for the enhancement of stability, and ultimately the treatment of wastewater. Although the rate-limiting stage differs and relies on the type of feed, the syntrophy activity is normally considered a key parameter that highly controls the whole rate of anaerobic digestion. Carbonaceous additives with high electrical conductivity enhance the syntrophy action involving acid generation by acidogenic and methane-producing microbes by IET, a concept involving both the DIET and IIET. In addition, the addition of conductive substances to the anaerobic digestion process could aid in stimulating the DIET method for microbes that are unable to generate nanowires for IET. Interestingly, the synergistic application of the bioelectrochemical system and anaerobic additives, especially carbonaceous additives such as magnetite-nanoparticles, is suggested as a very promising concept. This is because magnetite-nanoparticles aid the transfer of electrons to the electrode compartment by enhancing the electrical conductivity of the bacterial biofilm that grows on the electrode surface, and ultimately decreasing the opposition that ions and electrons encounter as they flow across a bioelectrochemical system. Therefore, this review demonstrated that the use of magnetite-nanoparticles and the bioelectrochemical system could be of significant use in addressing the challenges of the traditional anaerobic digestion process; however, additional research is necessary to fully comprehend this concept of wastewater treatment.

## Figures and Tables

**Table 1:** Inhibitory effect in anaerobic digestion [40].

Inhibitor	Impact on Anaerobic Digestion	Main Feedstock Waste	Reference
VFA (>200 mg/L)	Lower pH Unstable syntrophic reaction Reduced population of Achaea Sour digester	Foodstuff Sugar industry effluent	Capson-Tojo et al. [42]; Gerardi et al. [43]
D-Limonene (>2–3.5 gVS^−1^ day^−1^)	Higher cell permeability Cell destruction The lower population of microbes	Citrus waste and peelings	Fagbohungbe et al. [44]
Nitrogen (>3 g/L)	Proteins that are not balanced May hinder methanogen microorganisms Build-up of acids	Industrial effluent Urine of animals	Milán et al. [45]
Sulphur products (10–20 gVS/L·day)	Stimulates sulphur-reducing microorganisms that have competition with acetogenesis microorganisms Reduction of methanogenesis microorganisms	Abattoir Domestic fowl dung	Feng et al. [46]
High amounts of light and heavy metals (0.2–2 mM for Fe)	Disturbance of cells Inhibition of acetoclastic microorganisms Competition in cellular structure adsorption Limit cell formation This might result in the destabilization of the buffering capability Might include neutralization impact on the structure of cells	Industrial effluent Domestic	Glass and Orphan [47]
Aliphatic that are halogenated	Inhibits the methanogenic stage; COD removal reduced by 20% and VFA reduced by 509 mg/L Troubled energy of cells	Industry effluent Oil & grease	Liu et al. [48]; Kiser et al. [49]
Lignin	Resistance in anaerobic digestion Low production of methane Limited access to cellulose	Biomass for lignocellulose Harvest remains	Demirel and Scherer [50]

**Table 2:** Effect of biochemical conductive substrates for syntrophic activity.

Carbonaceous Additives	Effect in Anaerobic Digestion	Reference
Bio-char	Is able to increase the population of microorganisms and methane yield by more than 21 L/kg VS Helps in the mitigation of limonene Methyltransferases Mitigation of ammonia ion Reduces lag phase by 27–64% and enhanced the methanogenesis by 22–40%	Fagbohungbe et al. [58]; Wang et al. [59]
Activated carbon	Decreases digester delay time by 2 days Improves the DIET process and methane production by 17.4% Improves the consumption of acids	Capson-Tojo et al. [42]; Yang et al. [60]
Granular activated carbon	Help in increasing Firmicutes population with sludge reduction increase of 6.1% May tolerate low temperature Increased methane production of 13.1%	Yang et al. [60]; Peng et al. [61]
Carbon cloth	Higher methanogenesis activities Enabled 1.34-fold more organic loading than that of the control Volatile fatty acid utilization direct interspecies electron transfer	Lei et al. [62]
Magnetite	Enables the interspecies transfer between Archaea and microorganisms Improved bacterial variety in the digester	Wang et al. [63]

**Table 3:** Various additives used in anaerobic digestion.

Additive	Material/Type	Effect on Anaerobic Digestion	Reference
Syntrophic activity	Carbon cloth	Enhanced methane content by 10.1–23.0%	Feng et al. [66]
Metabolic activity	Iron (F^0^)	Highest removals of COD and phosphates were 88.0% and 98.0%, respectively.	Bakari et al. [70]
Catalytic activity	Cobalt and iron zero valent nanoparticles	Enhanced the early stages of the anaerobic digestion of waste-activated sludge.	Córdova-Lizama et al. [72]
Enzymatic activity	Carbon-based acids	Lactate, formate, and acetate have been found to function like promoters to improve the cellulosome activity for the duration of anaerobic digestion within 50, 100 and 200 mM concentrations, respectively.	Xu et al. [80]
Cation exchange activity	Zeolites	Increased the methane content by 19.7% and the overall methane yield by 120.9 CH_4_/kg VS.	Wang et al. [84]

**Table 4:** Previous studies on electrochemical efficiencies.

Type of Bioelectrochemical System	Effect of Electrochemical Efficiencies on Bioelectrochemical System	Reference
Microbial electrosynthesis system	Neutral pH achieved coulombic efficiency of over 200% High coulombic efficiency was evident of simultaneous EMY and HMY Increase in HMY resulted in an increase in methane content	Nelabhotla and Dinamarca [112]
Photo-Bioelectrochemical system	The bioelectrochemical system increased coulombic efficiency by 12.2% and degradation rate by 0.159 h^−1^	Wang et al. [113]
Microbial electrosynthesis system	An increase in current density resulted in an increase in electrical conductivity	Sleutels et al. [114]
MEC	Hydrogen was reduced to methane The high coulombic efficiency of 85 ± 2% was due to the high methane content of 76 ± 7%	Villano et al. [115]
MFC	Highest power density of 405 mW/m^2^ lead to COD removal of 82% and coulombic efficiency of 15%	Cordova-Bautista [116]

## Data Availability

Not applicable.

## References

[1] Saravanan A., Kumar P.S., Nhung T.C., Ramesh B., Srinivasan S., Rangasamy G. (2022). A review on biological methodologies in municipal solid waste management and landfilling: Resource and energy recovery. Chemosphere.

[2] Al-Dailami A., Ahmad I., Kamyab H., Abdullah N., Koti I., Veeramuthu A., Zabara B. (2022). Sustainable solid waste management in Yemen: Environmental, social aspects, and challenges. Biomass Conv. Bioref..

[3] Gielen D., Boshell F., Saygin D., Bazilian M.D., Wagner N., Gorini R. (2019). The role of renewable energy in the global energy transformation. Energy Strategy Rev..

[4] He M., Sun Y., Han B. (2021). Green Carbon Science: Efficient Carbon Resource Processing, Utilization, and Recycling towards Carbon Neutrality. Angew. Chem. Int. Ed..

[5] Ahmed A., Ge T., Peng J., Yan W.C., Tee B.T., You S. (2022). Assessment of the renewable energy generation towards net-zero energy buildings: A review. Energy Build..

[6] Rekleitis G., Haralambous K.-J., Loizidou M., Aravossis K. (2020). Utilization of Agricultural and Livestock Waste in Anaerobic Digestion (A.D): Applying the Biorefinery Concept in a Circular Economy. Energies.

[7] Kamperidou V., Terzopoulou P. (2021). Anaerobic Digestion of Lignocellulosic Waste Materials. Sustainability.

[8] Nabi M., Liang M., Cheng L., Yang W., Gao D. (2022). A comprehensive review on the use of conductive materials to improve anaerobic digestion: Focusing on landfill leachate treatment. J. Environ. Manag..

[9] Jadhav P., Muhammad N., Bhuyar P., Krishnan S., Razak A.S.A., Zularisam A.W., Nasrullah M. (2021). A review on the impact of conductive nanoparticles (CNPs) in anaerobic digestion: Applications and limitations. Environ. Technol. Innov..

[10] Wang W., Lee D.J., Lei Z. (2022). Integrating anaerobic digestion with microbial electrolysis cell for performance enhancement: A review. Bioresour. Technol..

[11] Madondo N.I., Kweinor Tetteh E., Rathilal S., Bakare B.F. (2022). Effect of an Electromagnetic Field on Anaerobic Digestion: Comparing an Electromagnetic System (ES), a Microbial Electrolysis System (MEC), and a Control with No External Force. Molecules.

[12] Xie J., Chang Y., Xie J., Adams M., Zhao D., Chen C., Ma J., Zhu G., Zhang T.C. (2021). Insights into the mechanism, performance and electrode modification of BES-AD combined systems for refractory wastewater treatment: A review. J. Water Process Eng..

[13] Wang B., Liu W., Liang B., Jiang J., Wang A. (2022). Microbial fingerprints of methanation in a hybrid electric-biological anaerobic digestion. Water Res..

[14] Fontanesi C., Kumar A., Mondal P.C. (2018). Overview on Induced Chirality in Magnetic Field Controlled Electro-Deposition and Induced Magnetic Moment Originating from Chiral Electrodes. Magnetochemistry.

[15] Madondo N.I., Tetteh E.K., Rathilal S., Bakare B.F. (2021). Synergistic Effect of Magnetite and Bioelectrochemical Systems on An-aerobic Digestion. Bioengineering.

[16] Madondo N.I., Tetteh E.K., Rathilal S., Bakare B.F. (2022). Application of Bioelectrochemical System and Magnetite Nanoparticles on the Anaerobic Digestion of Sewage Sludge: Effect of Electrode Configuration. Catalysts.

[17] Madondo N.I., Rathilal S., Bakare B.F. (2022). Utilization of Response Surface Methodology in Optimization and Modelling of a Microbial Electrolysis Cell for Wastewater Treatment Using Box–Behnken Design Method. Catalysts.

[18] Cruz Viggi C., Casale S., Chouchane H., Askri R., Fazi S., Cherif A., Zeppilli M., Aulenta F. (2019). Magnetite nanoparticles enhance the bioelectrochemical treatment of municipal sewage by facilitating the syntrophic oxidation of volatile fatty acids. J. Chem. Technol. Biotechnol..

[19] Mostafa A., Im S., Song Y.C., Ahn Y., Kim D.H. (2020). Enhanced Anaerobic Digestion by Stimulating DIET Reaction. Processes.

[20] El Mashad H.M., Barzee T.J., Franco R.B., Zhang R., Kaffka S., Mitloehner F. (2023). Anaerobic Digestion and Alternative Manure Management Technologies for Methane Emissions Mitigation on Californian Dairies. Atmosphere.

[21] Shapovalov Y., Zhadan S., Bochmann G., Salyuk A., Nykyforov V. (2020). Dry Anaerobic Digestion of Chicken Manure: A Review. Appl. Sci..

[22] Dalke R., Demro D., Khalid Y., Wu H., Urgun-Demirtas M. (2021). Current status of anaerobic digestion of food waste in the United States. Renew. Sustain. Energy Rev..

[23] Singh R., Paritosh K., Pareek N., Vivekanand V. (2022). Integrated system of anaerobic digestion and pyrolysis for valorization of agricultural and food waste towards circular bioeconomy: Review. Bioresour. Technol..

[24] Wang S., Xu C., Song L., Zhang J. (2022). Anaerobic Digestion of Food Waste and Its Microbial Consortia: A Historical Review and Future Perspectives. Int. J. Environ. Res. Public Health.

[25] Goswami L., Kushwaha A., Singh A., Saha P., Choi Y., Maharana M., Patil S.V., Kim B.S. (2022). Nano-Biochar as a Sustainable Catalyst for Anaerobic Digestion: A Synergetic Closed-Loop Approach. Catalysts.

[26] Kazimierowicz J., Dębowski M. (2022). Aerobic Granular Sludge as a Substrate in Anaerobic Digestion—Current Status and Perspectives. Sustainability.

[27] Ignatowicz K., Filipczak G., Dybek B., Wałowski G. (2023). Biogas Production Depending on the Substrate Used: A Review and Evaluation Study—European Examples. Energies.

[28] Kazimierowicz J., Dębowski M. (2023). Characteristics of Solidified Carbon Dioxide and Perspectives for Its Sustainable Application in Sewage Sludge Management. Int. J. Mol. Sci..

[29] Kesarwani S., Panwar D., Mal J., Pradhan N., Rani R. (2023). Constructed Wetland Coupled Microbial Fuel Cell: A Clean Technology for Sustainable Treatment of Wastewater and Bioelectricity Generation. Fermentation.

[30] Naphtali J., Chan A.W.Y., Saleem F., Li E., Devries J., Schellhorn H.E. (2022). Comparative Metagenomics of Anaerobic Digester Communities Reveals Sulfidogenic and Methanogenic Microbial Subgroups in Conventional and Plug Flow Residential Septic Tank Systems. Processes.

[31] Batstone D.J., Keller J., Angelidaki I., Kalyuzhnyi S.V., Pavlostathis S.G., Rozzi A., Sanders W.T.M., Siegrist H., Vavilin V.A. (2002). The IWA Anaerobic Digestion Model No 1(ADM1). Water Sci. Technol..

[32] Xu Z.X., Ma X.Q., Zhou J., Duan P.G., Zhou W.Y., Ahmad A., Luque R. (2022). The influence of key reactions during hydrothermal carbonization of sewage sludge on aqueous phase properties: A review. J. Anal. Appl. Pyrolysis.

[33] Uthirakrishnan U., Sharmila V.G., Merrylin J., Kumar S.A., Dharmadhas J.S., Varjani S., Banu J.R. (2022). Current advances and future outlook on pretreatment techniques to enhance biosolids disintegration and anaerobic digestion: A critical review. Chemosphere.

[34] Vázquez-Fernández A., Suárez-Ojeda M.E., Carrera J. (2022). Review about bioproduction of Volatile Fatty Acids from wastes and wastewaters: Influence of operating conditions and organic composition of the substrate. J. Environ. Chem. Eng..

[35] Das N., Jena P.K., Padhi D., Mohanty M.K., Sahoo G. (2023). A comprehensive review of characterization, pretreatment and its applications on different lignocellulosic biomass for bioethanol production. Biomass Conv. Bioref..

[36] Prasad B.R., Padhi R.K., Ghosh G. (2022). A review on key pretreatment approaches for lignocellulosic biomass to produce biofuel and value-added products. Int. J. Environ. Sci. Technol..

[37] Olatunji K.O., Ahmed N.A., Ogunkunle O. (2021). Optimization of biogas yield from lignocellulosic materials with different pretreatment methods: A review. Biotechnol. Biofuels.

[38] Dahiya S., Lingam Y., Mohan S.V. (2023). Understanding acidogenesis towards green hydrogen and volatile fatty acid production—Critical Analysis and Circular Economy Perspective. Chem. Eng. J..

[39] Dong W., Yang Y., Liu C., Zhang J., Pan J., Luo L., Wu G., Awasthi M.K., Yan B. (2023). Caproic acid production from anaerobic fermentation of organic waste—Pathways and microbial perspective. Renew. Sustain. Energy Rev..

[40] Paritosh K., Yadav M., Chawade A., Sahoo D., Kesharwani N., Pareek N., Vivekanand V. (2020). Additives as a Support Structure for Specific Biochemical Activity Boosts in Anaerobic Digestion: A Review. Front. Energy Res..

[41] Anukam A., Mohammadi A., Naqvi M., Granstrom K. (2019). A review of the Chemistry of Anaerobic Digestion: Methods of Accelerating and Optimizing Process Efficiency. Processes.

[42] Capson-Tojo G., Moscoviz R., Ruiz D., Santa-Catalina G., Trably E., Rouez M., Crest M., Steyer J.P., Bernet N., Delgenes J.P. (2018). Addition of granular activated carbon and trace elements to favor volatile fatty acid consumption during anaerobic digestion of food waste. Bioresour. Technol..

[43] Gerardi M.H. (2006). Wastewater Bacteria.

[44] Fagbohungbe M.O., Herbert B.M.J., Hurst L., Ibeto C.N., Li H., Usmani S.Q., Semple K.T. (2017). The challenges of anaerobic digestion and the role of biochar in optimizing anaerobic digestion. Waste Manag..

[45] Milán Z., Montalvo S., Ilangovan K., Monroy O., Chamy R., Weiland P., Sanchez E., Borja R. (2010). The impact of ammonia nitrogen concentration and zeolite addition on the specific methanogenic activity of granular and flocculent anaerobic sludges. J. Environ. Sci. Health Part A.

[46] Feng X.M., Karlsson A., Svensson B.H., Bertilsson S. (2010). Impact of trace element addition on biogas production from food industrial waste–linking process to microbial communities. FEMS Microbiol. Ecol..

[47] Glass J., Orphan V.J. (2012). Trace metal requirements for microbial enzymes involved in the production and consumption of methane and nitrous oxide. Front. Microbiol..

[48] Liu Y., Zhang Y., Quan X., Li Y., Zhao Z., Meng X., Chen S. (2012). Optimization of anaerobic acidogenesis by adding Fe0 powder to enhance anaerobic wastewater treatment. Chem. Eng. J..

[49] Kiser M.A., Ryu H., Jang H., Hristovski K., Westerhoff P. (2010). Biosorption of nanoparticles to heterotrophic wastewater biomass. Water Res..

[50] Demirel B., Scherer P. (2011). Trace element requirements of agricultural biogas digesters during biological conversion of renewable biomass to methane. Biomass Bioenergy.

[51] Liu M., Wei Y., Leng X. (2021). Improving biogas production using additives in anaerobic digestion: A review. J. Clean. Prod..

[52] Liu C., Tong Q., Li Y., Wang N., Liu B., Xhang X. (2019). Biogas production and metal passivation analysis during anaerobic digestion of pig manure: Effects of a magnetic Fe_3_O_4_/FA composite supplement. R. Soc. Chem..

[53] Xu X.J., Yan J., Yuan Q.K., Wang X.T., Yuan Y., Ren N.Q., Lee D.J., Chen C. (2022). Enhanced methane production in anaerobic digestion: A critical review on regulation based on electron transfer. Bioresour. Technol..

[54] Castilho T.G., Rodrigues J.A.D., García J., Subtil E.L. (2022). Recent advances and perspectives in the use of conductive materials to improve anaerobic wastewater treatment: A systematic review approached. J. Water Process Eng..

[55] González J., Sánchez M.E., Gómez X. (2018). Enhancing Anaerobic Digestion: The Effect of Carbon Conductive Materials. C.

[56] Lee C., Sinharoy A., Lens P.N.L. (2022). Engineering Direct Interspecies Electron Transfer for Enhanced Methanogenic Performance. Renewable Energy Technologies for Energy Efficient Sustainable Development.

[57] Li L., Xu Y., Dai X., Dai L. (2021). Principles and advancements in improving anaerobic digestion of organic waste via direct interspecies electron transfer. Renew. Sustain. Energy Rev..

[58] Fagbohungbe M.O., Herbert B.M., Hurst L., Li H., Usmani S.Q., Semple K.T. (2016). Impact of biochar on the anaerobic digestion of citrus peel waste. Bioresour. Technol..

[59] Wang G., Li Q., Gao X., Wang X.C. (2018). Synergetic promotion of syntrophic methane production from anaerobic digestion of complex organic wastes by biochar: Performance and associated mechanisms. Bioresour. Technol..

[60] Yang Y., Zhang Y., Li Z., Zhao Z., Quan X., Zhao Z. (2017). Adding granular activated carbon into anaerobic sludge digestion to promote methane production and sludge decomposition. J. Clean. Prod..

[61] Peng H., Zhang Y., Tan D., Zhao Z., Zhao H., Quan X. (2018). Roles of magnetite and granular activated carbon in improvement of anaerobic sludge digestion. Bioresour. Technol..

[62] Lei Y., Sun D., Dang Y., Chen H., Zhao Z., Zhang Y., Holmes D.E. (2016). Stimulation of methanogenesis in anaerobic digesters treating leachate from a municipal solid waste incineration plant with carbon cloth. Bioresour. Technol..

[63] Wang Z.K., Liu Q.H., Yang Z.M. (2023). Nano magnetite-loaded biochar boosted methanogenesis through shifting microbial community composition and modulating electron transfer. Sci. Total Environ..

[64] Park J.H., Kang H.J., Park K.H., Park H.D. (2018). Direct interspecies electron transfer via conductive materials: A perspective for anaerobic digestion applications. Bioresour. Technol..

[65] Shin D.C., Kim I.-T., Jung J., Jeong Y., Lee Y.-E., Ahn K.-H. (2022). Increasing Anaerobic Digestion Efficiency Using Food-Waste-Based Biochar. Fermentation.

[66] Feng D., Xia A., Huang Y., Zhu X., Zhu X., Liao Q. (2022). Effects of carbon cloth on anaerobic digestion of high concentration organic wastewater under various mixing conditions. J. Hazard. Mater..

[67] Mostafa A., Im S., Song Y.-C., Kang S., Kim D.-H. (2020). Enhanced Anaerobic Digestion of Long Chain Fatty Acid by Adding Magnetite and Carbon Nanotubes. Microorganisms.

[68] Paulo L., Stams A., Sousa D. (2015). Methanogens, sulphate and heavy metals: A complex system. Rev. Environ. Sci. Biotechnol..

[69] Zhang H., Tian Y., Zheng L., Li S., Hao H., Yin M., Cao Y., Huang H. (2019). Process Analysis of Anaerobic Fermentation Exposure to Metal Mixtures. Int. J. Environ. Res. Public Health.

[70] Bakari O., Njau K.N., Noubactep C. (2022). Fe0-Supported Anaerobic Digestion for Organics and Nutrients Removal from Domestic Sewage. Water.

[71] Rahman K., Melville S., Imamul Huq S., Khoda S. (2016). Understanding bioenergy production and optimisation at the nanoscale—A review. J. Exp. Nanosci..

[72] Córdova-Lizama A., Carrera-Figueiras C., Palacios A., Castro-Olivera P.M., Ruiz-Espinoza J. (2022). Improving hydrogen production from the anaerobic digestion of waste activated sludge: Effects of cobalt and iron zero valent nanoparticles. Int. J. Hydrog. Energy.

[73] García A., Delgado L., Torà J.A., Casals E., González E., Puntes V., Font X., Carrera J., Sánchez A. (2012). Effect of cerium dioxide, titanium dioxide, silver, and gold nanoparticles on the activity of microbial communities intended in wastewater treatment. J. Hazard. Mater..

[74] Chhetri T., Cunningham G., Suresh D., Shanks B., Kannan R., Upendran A., Afrasiabi Z. (2022). Wastewater Treatment Using Novel Magnetic Nanosponges. Water.

[75] Award E.S., Sabirova T.M., Tretyakova N.A., Alsalhy Q.F., Figoli A., Salih I.K. (2021). A Mini-Review of Enhancing Ultrafiltration Membranes (UF) for Wastewater Treatment: Performance and Stability. Chem. Eng..

[76] Kaegi R., Voegelin A., Sinnet B., Zuleeg S., Hagendorfer H., Burkhardt M., Siegrist H. (2011). Behavior of Metallic Silver Nanoparticles in a Pilot Wastewater Treatment Plant. Environ. Sci. Technol..

[77] Li Y.Z., Inoue D., Ike M. (2023). Mitigating ammonia-inhibition in anaerobic digestion by bioaugmentation: A review. J. Water Process Eng..

[78] Parawira W. (2012). Enzyme research and applications in biotechnological intensification of biogas production. Crit. Rev. Biotechnol..

[79] Heerenklage J., Rechtenbach D., Atamaniuk I., Alassali A., Raga R., Koch K. (2019). Development of a method to produce standardised and storable inocula for biomethane potential tests–preliminary steps. Renew. Energy.

[80] Xu C., Qin Y., Li Y., Ji Y., Huang J., Song H. (2010). Factors influencing cellulosome activity in consolidated bioprocessing of cellulosic ethanol. Bioresour. Technol..

[81] Yu C., Li D., Wang Q., Zhang Z., Yang Y. (2014). Improving Anaerobic Methane Production from Ammonium-rich Piggery Waste in a Zeolite-fixed Bioreactor and Evaluation of Ammonium Adsorbed on Zeolite A-3 as Fertilizer. Int. J. Waste Resour..

[82] Ciezkowska M., Bajda T., Decewicz P., Dziewit D., Drewniak L. (2020). Effect of Clinoptilolite and Halloysite Addition on Biogas Production and Microbial Community Structure during Anaerobic Digestion. Materials.

[83] Lauka D., Pastare L., Blumberga D., Romagnoli F. Preliminary analysis of anaerobic digestion process using *Cerathophyllum demersum* and low carbon content additives: A batch test study. Proceedings of the International Scientific Conference “Environmental and Climate Technologies—CONECT 2014”.

[84] Wang Q., Yang Y., Yu C., Huang H., Kim M., Feng C., Zhang Z. (2011). Study on a fixed zeolite bioreactor for anaerobic digestion of ammonium-rich swine wastes. Bioresour. Technol..

[85] Hansson A. (2011). Mechanism of Zeolite Activity in Biogas Co-Digestion. Master’s Thesis.

[86] Zhang Y., Li C., Yuan Z., Wang R., Angelidaki I., Zhu G. (2023). Syntrophy mechanism, microbial population, and process optimization for volatile fatty acids metabolism in anaerobic digestion. Chem. Eng. J..

[87] Hamelers H.M., Heijne A., Sleutels T.J.A., Jeremiasse A., Strik D.B.T.B., Buisman C.N. (2010). New applications and performance of bioelectrochemical systems. Appl. Microbiol. Biotechnol..

[88] Zhang X., Li X., Zhao X., Li Y. (2019). Factors affecting the efficiency of a bioelectrochemical system: A review. RSC Adv..

[89] Pant D., Singh A., Van Bogaert G., Irving Olsen S., Singh Nigam P., Diels L., Vanbroekhoven K. (2012). Bioelectrochemical systems (BES) for sustainable energy production and product recovery from organic wastes and industrial wastewaters. RSC Adv..

[90] Vijay A., Sonawane J.M., Chhabra M. (2022). Denitrification process in microbial fuel cell: A comprehensive review. Bioresour. Technol. Rep..

[91] Gorby Y.U., Yanina S., McLean J.S., Rosso K.M., Moyles D., Dohnalkova A., Beveridge T.J., Chang I.S., Kim B.H., Culley D.E. (2006). Electrically conductive bacterial nanowires produced by *Shewanella oneidensis* strain MR-1 and other microorganisms. Proc. Natl. Acad. Sci. USA.

[92] Kim I.S., Chae K., Choi M., Vestraete W. (2008). Microbial Fuel Cells: Recent advances, bacterial communities and application beyond electricity generation. Environ. Eng. Res..

[93] Aiswaria P., Mohamed S.N., Singaravelu D.L., Brindhadevi K., Pugazhendhi A. (2022). A review on graphene/graphene oxide supported electrodes for microbial fuel cell applications: Challenges and prospects. Chemosphere.

[94] Villano M., Aulenta F., Majone M. (2012). Perspectives of biofuels production from renewable resources with bioelectrochemical systems. Asia Pac. J. Chem. Eng..

[95] Hassan M., Zhu G., Lu Y., Al-Falahi A.H., Lu Y., Huang S., Wan Z. (2021). Removal of antibiotics from wastewater and its problematic effects on microbial communities by bioelectrochemical Technology: Current knowledge and future perspectives. Environ. Eng. Res..

[96] Tzelepis S., Kavadias K.A., Marnellos G.E., Xydis G. (2021). A review study on proton exchange membrane fuel cell electrochemical performance focusing on anode and cathode catalyst layer modelling at macroscopic level. Renew. Sustain. Energy Rev..

[97] Prathiba S., Kumar P.S., Vo D.V.N. (2022). Recent advancements in microbial fuel cells: A review on its electron transfer mechanisms, microbial community, types of substrates and design for bio-electrochemical treatment. Chemosphere.

[98] Radhika D., Shivakumar A., Kasai D.R., Koutavarapu R., Peera S.G. (2022). Microbial Electrolysis Cell as a Diverse Technology: Overview of Prospective Applications, Advancements, and Challenges. Energies.

[99] Rabaey K., Verstraete W. (2005). Microbial fuel cells: Novel biotechnology for energy generation. Trends Biotechnol..

[100] Chatterjee P., Dessì P., Kokko M., Lakaniemi A.M., Lens P. (2019). Selective enrichment of biocatalysts for bioelectrochemical systems: A critical review. Renew. Sustain. Energy Rev..

[101] Hassanein A., Witarsa F., Lansing S., Qiu L., Liang Y. (2020). Bio-Electrochemical Enhancement of Hydrogen and Methane Production in a Combined Anaerobic Digester (AD) and Microbial Electrolysis Cell (MEC) from Dairy Manure. Sustainability.

[102] An Z., Feng Q., Zhao R., Wang X. (2020). Bioelectrochemical Methane Production from Food Waste in Anaerobic Digestion Using a Carbon-Modified Copper Foam Electrode. Processes.

[103] Li S., Chen G. (2018). Factors Affecting the Effectiveness of Bioelectrochemical System Applications: Data Synthesis and Meta-Analysis. Batteries.

[104] Tang Y.L., He Y.T., Yu P.F., Sun H., Fu J.X. (2012). Effect of temperature on electricity generation of single-chamber microbial fuel cells with proton exchange membrane. Adv. Mater. Res..

[105] Trinh N.T., Park J.H., Kim B.W. (2009). Increased generation of electricity in a microbial fuel cell using geobacter sulfurreducens. Korean J. Chem. Eng..

[106] Liu Y., Climent V., Berna A., Feliu J.M. (2011). Effect of temperature on the catalytic ability of electrochemically active biofilm as anode catalyst in microbial fuel cells. Electroanalysis.

[107] Li L.H., Sun Y.M., Yuan Z.H., Kong X.Y., Li Y. (2013). Effect of temperature change on power generation of microbial fuel cell. Environ. Technol..

[108] Liu H., Cheng S.A., Logan B.E. (2005). Power generation in fed-batch microbial fuel cells as a function of ionic strength, temperature, and reactor configuration. Environ. Sci. Technol..

[109] Heidrich E.S., Dolfing J., Wade M., Sloan W.T., Quince C., Curtis T.P. (2018). Temperature, inocula and substrate: Contrasting electroactive consortia, diversity and performance in microbial fuel cells. Bioelectrochemistry.

[110] Ahn Y., Im S., Chung J.W. (2017). Optimizing the operating temperature for microbial electrolysis cell treating sewage sludge. Int. J. Hydrogen Energy.

[111] Xia Y., Wang G., Guo L., Dai Q., Ma X. (2020). Electrochemical oxidation of Acid Orange 7 azo dye using a PbO2 electrode: Parameter optimization, reaction mechanism and toxicity evaluation. Chemosphere.

[112] Nelabhotla A.B.T., Dinamarca C. (2019). Bioelectrochemical CO_2_ Reduction to Methane: MES Integration in Biogas Production Processes. Appl. Sci..

[113] Wang Y., Gan L., Liao Z., Hou R., Zhou S., Zhou l., Yuan Y. (2022). Self-produced biophotosensitizers enhance the degradation of organic pollutants in photo-bioelectrochemical systems. J. Hazard. Mater..

[114] Sleutels T.H.J.A., Molenaar S.D., Ter Heijne A., Buisman C.J.N. (2016). Low Substrate Loading Limits Methanogenesis and Leads to High Coulombic Efficiency in Bioelectrochemical Systems. Microorganisms.

[115] Villano M., Aulenta F., Ciucci C., Ferri T., Giuliano A., Majone M. (2010). Bioelectrochemical reduction of CO_2_ to CH_4_ via direct and indirect extracellular electron transfer by a hydrogenophilic methanogenic culture. Bioresour. Technol..

[116] Cordova-Bautista Y., Paraguay-Delgado F., Perez Hernandez B., Perez Hernandez G., Martinez Pereyra G., Ramirez Morales E. (2018). Influence of external resistance and anodic pH on power density in microbial fuel cell operated with *B. subtilis* BSC-2 strain. Appl. Ecol. Environ. Res..

[117] Konopacki M., Rakoczy R. (2019). The analysis of rotating magnetic field as a trigger of Gram-positive and Gram-negative bacteria growth. Biochem. Eng. J..

[118] Nopharatana A., Pullammanappallil P.C., Clarke W.P. (2007). Kinetic and dynamic modelling of batch anaerobic digestion of municipal solid waste in a stirred reactor. Waste Manag..

[119] Mersinkova Y., Yemendzhiev H., Nenov V. (2022). Comparative study on the metabolic behaviour of anode biofilm in microbial fuel cell under different external resistance. Biotechnol. Biotechnol. Equip..

[120] Kamau J.M., Mbui D.N., Mwaniki J.M., Mwaura F.B., Kamau G.N. (2017). Microbial Fuel Cells: Influence of External Resistors on Power, Current and Power Density. J. Thermodyn. Catal..

[121] Chen Q., Liu C., Liu X., Sun D., Li P., Qiu P., Dang Y., Karpinski N.A., Smith J.A., Holmes D.E. (2020). Magnetite enhances anaerobic digestion of high salinity organic wastewater. Environ. Res..

[122] Cavalcante W.A., Gehring T.A., Zaiat M. (2021). Stimulation and inhibition of direct interspecies electron transfer mechanisms within methanogenic reactors by adding magnetite and granular actived carbon. Chem. Eng. J..

[123] Wang R., Li H., Sun J., Zhang L., Jiao J., Wang Q., Liu S. (2021). Nanomaterials Facilitating Microbial Extracellular Electron Transfer at Interfaces. Adv. Mater..

[124] Vu M.T., Noori M.T., Min B. (2020). Magnetite/zeolite nanocomposite-modified cathode for enhancing methane generation in microbial electrochemical systems. Chem. Eng. J..

[125] Hamed M.S., Majdi H., Hasan B.O. (2020). Effect of Electrode Material and Hydrodynamics on Produced Current in Double Chamber Microbial Fuel Cells. ACS Omega.

[126] Qin X., Lu X., Cai T., Niu C., Han Y., Zhang Z., Zhu X., Zhen G. (2021). Magnetite-enhanced bioelectrochemical stimulation for biodegradation and biomethane production of waste activated sludge. Sci. Total Environ..

